# Relational factors predict telepsychotherapy acceptance in patients: The role of therapeutic relationship and attachment

**DOI:** 10.1192/j.eurpsy.2022.448

**Published:** 2022-09-01

**Authors:** V. Békés, K. Aafjes-Van Doorn

**Affiliations:** Yeshiva University, Ferkauf Graduate School Of Psychology, Bronx, United States of America

**Keywords:** attitude, patient, Covid-19, Telepsychotherapy

## Abstract

**Introduction:**

During the early months of the COVID-19 pandemic, patients receiving individual psychotherapy needed to transition to telepsychotherapy (TP). Since telemental health appears to be here to stay after the pandemic ends, it is crucial to understand factors that determine whether telemental health is a good fit for patients.

**Objectives:**

The aim of the present study was to (1) explore patients’ perception of the therapeutic relationship and attitudes towards TP, and (2) identify predictors of patients’ TP acceptance.

**Methods:**

We used a longitudinal design, where patients (N = 719) receiving individual TP during the pandemic participated in an online survey, in which they responded to demographic questions and completed measures of symptom severity, Covid-related distress, attachment style (avoidant/anxious), perceived quality of the therapeutic relationship (working alliance and real relationship), and TP acceptance.

**Results:**

We found that (1) patients perceived the quality of the therapeutic relationship as reasonably good, and patients’ TP acceptance was moderately high. (2) patients’ TP acceptance was predicted by their attachment avoidance and their perception of the real relationship, whereas attachment anxiety, working alliance, as well as demographic variables, symptom severity, and Covid-related distress were unrelated to TP acceptance. The final model showed that perceived strength of the real relationship mediated the relationship between attachment avoidance and TP acceptance.

**Conclusions:**

Both general (attachment) and situational (therapeutic relationship) relational variables are important predictors of patient’s acceptance of TP, and should be considered during decision making about suitability of TP to patients.

**Disclosure:**

No significant relationships.

